# Psychosocial factors are independent risk factors for the development of Type 2 diabetes in Japanese workers with impaired fasting glucose and/or impaired glucose tolerance^1^

**DOI:** 10.1111/j.1464-5491.2008.02566.x

**Published:** 2008-10

**Authors:** M Toshihiro, K Saito, S Takikawa, N Takebe, T Onoda, J Satoh

**Affiliations:** Department of Diabetes and Metabolism, Iwate Medical UniversityMorioka, Iwate, Japan; * Nishimatsuzono Clinic for Internal MedicineMorioka, Iwate, Japan; † Department of Public Health, Iwate Medical UniversityMorioka, Iwate, Japan

**Keywords:** impaired fasting glucose, impaired glucose tolerance, psychosocial factors, Type 2 diabetes

## Abstract

**Aims:**

We prospectively studied Japanese workers with impaired fasting glucose (IFG) and/or impaired glucose tolerance (IGT) and analysed possible risk factors for diabetes, including psychosocial factors such as stress.

**Methods:**

The participants were 128 male Japanese company employees (mean age, 49.3 ± 5.9 years) with IFG and/or IGT diagnosed by oral glucose tolerance test (OGTT). Participants were prospectively studied for 5 years with annual OGTTs. The Kaplan–Meier method and Cox's proportional hazard model were used to analyse the incidence of diabetes and the factors affecting glucose tolerance, including anthropometric, biochemical and social–psychological factors.

**Results:**

Of 128 participants, 36 (28.1%) developed diabetes and 39 (30.5%) returned to normal glucose tolerance (NGT) during a mean follow-up of 3.2 years. Independent risk factors for diabetes were night duty [hazard ratio (HR) = 5.48, *P* = 0.002], higher fasting plasma glucose (FPG) levels within 6.1–6.9 mmol/l (HR = 1.05, *P* = 0.031), stress (HR = 3.81, *P* = 0.037) and administrative position (HR = 12.70, *P* = 0.045), while independent factors associated with recovery were lower FPG levels (HR = 0.94, *P* = 0.017), being a white-collar worker (HR = 0.34, *P* = 0.033), non-smoking (HR = 0.31, *P* = 0.040) and lower serum alanine aminotransferase (ALT) levels (HR = 0.97, *P* = 0.042).

**Conclusions:**

In addition to FPG levels at baseline, psychosocial factors (night duty, stress and administrative position) are risk factors for Type 2 diabetes, while being a white-collar worker, a non-smoker and lower serum ALT levels are factors associated with return to NGT in Japanese workers with IFG and/or IGT.

## Introduction

Over recent decades, the prevalence of Type 2 diabetes and pre-diabetes has been increasing both worldwide and in Japan. Risk factors for the development of Type 2 diabetes have been studied to facilitate prediction and prevention of this condition. By analysing anthropometric measurements and blood biochemistry markers, obesity [1,2] and elevated liver enzymes [3,4] have been reported as such risk factors. Increased energy intake as a result of a high-fat diet and decreased energy expenditure as a result of a sedentary lifestyle have resulted in obesity, which carries a high risk for the development of diabetes [1,2]. Increased alanine aminotransferase (ALT), a marker of hepatocyte damage, is strongly correlated with obesity, fatty liver, insulin resistance, Type 2 diabetes and the metabolic syndrome [3,4]. Gamma-glutamyl transpeptidase (γ-GTP) is also associated with the metabolic syndrome and Type 2 diabetes [5].

In addition, psychological factors such as stress have also been implicated in the development of Type 2 diabetes [6]. There have been a number of large-scale cross-sectional studies investigating stress and Type 2 diabetes [7,8]. In healthy Swedish women, work stress, which was defined as situations with high demands and low decision latitude, was associated with insulin resistance and diabetes [7]. Stressful life events were marginally correlated with the prevalence of undetected diabetes in the general population [8]. However, there might be a bidirectional association between stress and diabetes, as recently reported in a study on depressive symptoms [9]. However, recently, several longitudinal studies have shown a relationship between psychological features and development of diabetes [10,11], as already reported with cardiovascular diseases [12,13]. A prospective study in healthy women revealed that stressful life events predict the development of the metabolic syndrome, a risk factor for the subsequent development of Type 2 diabetes [10]. A case–referent study has also shown that work stress and low emotional support is associated with increased risk of future Type 2 diabetes in women [11].

Thus, a variety of risk factors affect the development of Type 2 diabetes in the general population. In addition, impaired fasting glucose (IFG) and/or impaired glucose tolerance (IGT) carry the greatest risk for the development of Type 2 diabetes [14,15]. Approximately half of those with IFG and/or IGT develop diabetes within 10 years and lifestyle interventions, such as dietary and exercise therapy, reduce the development of diabetes from IFG and/or IGT with obesity by more than 50%[16,17].

Because subjects with IFG and/or IGT are the most important population to target for the prevention of Type 2 diabetes, a variety of factors that may be associated with progression or regression of IFG and/or IGT need to be clarified. In the present study, to comprehensively and simultaneously analyse possible risk factors, we prospectively analysed social factors (position within the workplace and working conditions) and psychological factors (stress in daily life, satisfaction with lifestyle and personal characteristics), as well as anthropometric measurements and blood biochemistry, for associations with progression to Type 2 diabetes mellitus (DM) and with the recovery of normal glucose tolerance (NGT) from IFG and/or IGT in Japanese male workers. We used questionnaires to determine stress in daily life and satisfaction with lifestyle in Japanese, developed and validated for Japanese people [18].

## Methods

### Subjects

Of 732 male workers of one specific railroad company who received a health check at Nishimatsuzono Clinic between 2000 and 2005, 128 men (mean age of 49.3 ± 5.9 years) were diagnosed with IFG (*n* = 14) and/or IGT (*n* = 114) by the American Diabetes Association's criteria (1997) [19] using a 75-g oral glucose tolerance test (OGTT). The participants with IFG and/or IGT were given advice about lifestyle modifications once or twice a year; for example, dietary and exercise therapy and body weight reduction of more than 5% if obese. An annual OGTT was carried out during the observation period (mean observation period of 3.2 ± 0.1 years) and progression to DM or recovery of NGT from IFG and/or IGT at baseline was determined. Those with type B or C hepatitis virus infections were excluded. The study was approved by the ethics committee of Iwate Medical University and the investigation was performed in accordance with the guidelines of the Declaration of Helsinki.

### OGTT and definition of glucose tolerance

NGT was defined as a fasting plasma glucose (FPG) level < 6.1 mmol/l and a 2-h plasma glucose level < 7.8 mmol/l. IFG was defined as an FPG level of 6.1–6.9 mmol/l and a 2-h plasma glucose level < 7.8 mmol/l. IGT was defined as an FPG level < 7.0 mmol/l and a 2-h plasma glucose level of 7.8–11.1 mmol/l. Type 2 diabetes was defined as a fasting glucose level ≥ 7.0 mmol/l, a 2-h plasma glucose level > 11.1 mmol/l or a non-fasting plasma glucose level > 11.1 mmol/l [19].

### Anthropometric measurements and blood biochemistry examination

Height, weight, body mass index (BMI), body fat mass, systolic blood pressure, diastolic blood pressure and blood chemistry were measured once a year. Blood was collected after an overnight fast of at least 12 h and used for measurement of plasma glucose (PG), glycated haemoglobin (HbA_1c_), aspartate transaminase (AST), ALT, γ-GTP, lactic dehydrogenase (LDH), alkaline phosphatase (ALP), total protein (TP), total cholesterol (TC), triglycerides (TG), LDL cholesterol (LDL-C), HDL cholesterol (HDL-C), uric acid (UA), amylase, creatinine (Cr), red blood cells (RBC), white blood cells (WBC), haemoglobin (Hb), haematocrit (Ht) and erythrocyte sedimentation rate (ESR). Morning urine was collected to measure urinary protein and proteinuria was defined as > 300 mg/l. Habitual alcohol drinking and current smoking were also determined by a questionnaire.

A determination of the presence of fatty liver was made by ultrasound examination [20]. Body fat (%) was measured using an impedance method (TANITA body fat analyzer; Tanita Corp., Tokyo, Japan).

### Analysis of social and psychological factors

Position at work (administrative or non-administrative) and working conditions (daytime service or night duty, blue-collar or white-collar work, and being a business bachelor, who lives alone in a separate town from his family for his new position, or not) were assessed for each participant by questionnaires. Stress in daily life and satisfaction with lifestyle were scored by the questionnaires developed for Japanese people by Ono and Nakamura [18] based on the study of Berne [21]. Briefly, participants were classified as experiencing stress if they answered ‘yes’ to more than five out of the following 15 questions: (1) You have no particular difficulty but feel impatient somehow. (2) You feel that this is not supposed to be. (3) You feel that you cannot keep up with other people. (4) You are very concerned about things that other people are doing. (5) You always have a vague feeling of unrest. (6) You have no sense of fulfillment in daily life. (7) You feel that you are always hurried by something. (8) You feel that you have something else to do. (9) You often behave according to the surrounding circumstances. (10) You are not clearly aware of your true feelings. (11) You have no confidence in your behaviour. (12) You experience malaise and lack energy. (13) You are apt to think about things negatively. (14) You have nothing to live for. (15) You often sigh.

Similarly, satisfaction with lifestyle was given a score based on the number of questions to which a ‘yes’ answer was given out of the following 15 questions: (1) You can express your feeling effectively. (2) Your behaviour takes into account your situation. (3) You make an effort but do not attempt to do things beyond your power. (4) You clearly recognize and can explain your image. (5) When you express your feelings to another person, you consider how you would feel if you were in that person's position. (6) You are very aware of your likes and dislikes but do not stick to them. (7) You do not have a particular schedule for eating and sleeping and live life your own way. (8) You have a sound understanding of your good and bad points, including your ideal weight. (9) You are able to understand changes and are able to manage them calmly. (10) You are able to think positively. (11) You have a sense of fulfillment after vigorous activity. (12) You are emotionally expressive. (13) You are emotionally stable. (14) You can recover in a relatively short time if depressed. (15) You have a person who will help you when you experience difficulties and you can express these to him/her.

The validity of the stress questionnaire has already been determined in 592 Japanese subjects (185 males and 407 females, average 44.8 years old) [18]. The Pearson correlation coefficients between the score of the stress questionnaire and the manifest anxiety scale (MAS) and the self-rating depression scale (SDS) were 0.52 (*P* < 0.01) and 0.49 (*P* < 0.01), respectively. Item-remainder correlation coefficient and internal consistency were also assessed. The item-remainder correlation coefficient was 0.56 to 0.81 and the Cronbach's α was 0.80. Similarly, the Cronbach's α for the lifestyle satisfaction questionnaire was 0.73. Based on these results, we believe that these questionnaires for stress in daily life and satisfaction with lifestyle are valid. Internal validity of the stress questionnaires and the lifestyle satisfaction questionnaire was confirmed by factor analysis using SPSS (SPSS Japan Inc., Tokyo, Japan).

Personality characteristics were analysed using an Egogram Check List [22]: CP (Critical Parent), NP (Nurturing Parent), A (Adult), FC (Free Child) and AC (Adapted Child). Cronbach's α was 0.57 for CP, 0.55 for NP, 0.60 for A, 0.61 for FC and 0.75 for AC, respectively. Egogram (CP > NP) is a CP-dominant group that includes persons who criticize others. Egogram (AC > FC) is an AC-dominant group that includes persons who try to adapt to other persons [23].

### Statistical analyses

Progression to DM and recovery of NGT from IFG and/or IGT at baseline were analysed using the Kaplan–Meier method. A multivariate analysis of the respective, independent risk factors and recovery factors was performed by Cox's proportional-hazards model using Dr. SPSS II (SPSS Japan Inc.).

Participants were classified into three groups: those with recovery of NGT from IFG and/or IGT at baseline during the observation period (‘Recovered’), those with persistent IFG and/or IGT (‘Persistent’) and those with progression to DM from IFG and/or IGT (‘DM’). The baseline anthropometric measurements, blood biochemistry factors and social and psychological factors were compared between two groups at a time; i.e. ‘Persistent’ vs. ‘DM’, ‘Persistent’ vs. ‘Recovered’ and ‘Recovered’ vs. ‘DM’, using the Mann–Whitney test or χ^2^-test by Dr. SPSS II.

## Results

**Figure 1:**
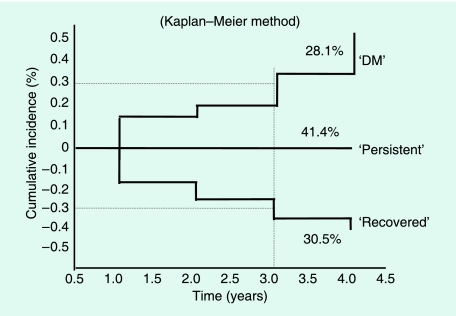
Change in status of glucose tolerance from impaired fasting glucose (IFG) and/or impaired glucose tolerance (IGT).

The proportion of participants who progressed to diabetes from IFG and/or IGT was 28.1% (36/128), whereas in 41.4% (53/128) of participants, IFG and/or IGT persisted and 30.5% (39/128) of participants with IFG and/or IGT reverted to NGT, during a mean observation period of 3.2 years on analysis using the Kaplan–Meier method (Fig. 1). Next, univariate analyses were performed using the Mann–Whitney and χ^2^ methods to compare various factors between the three groups, i.e. ‘Recovered’, ‘Persistent’ and ‘DM’. In comparison with the ‘Persistent’ group, ESR, FPG and frequency of blue-collar workers were significantly higher in the ‘DM’ group and the prevalence of blue-collar workers was lower in the ‘Recovered’ group. ALP, ESR, FPG and prevalence of business bachelor were significantly higher in the ‘DM’ group than those in the ‘Recovered’ group (Table 1).

**Table 1:** Clinical characteristics of participants with IFG and/or IGT at baseline according to change in glucose tolerance during the observation period

Characteristics	‘Recovered’	‘Persistent’	‘DM’
No. of participants	39	53	36
Age (years)	47.7 ± 6.4	49.6 ± 5.6	50.5 ± 5.8
Height (cm)	167.6 ± 5.6	168.4 ± 4.6	168.1 ± 5.8
Weight (kg)	69.7 ± 7.4	70.1 ± 8.7	70.4 ± 9.2
Body fat (%)	25.4 ± 4.4	25.5 ± 5.6	25.2 ± 6.2
Body mass index (kg/m^2^)	24.8 ± 2.3	24.5 ± 5.6	24.9 ± 3.3
Systolic blood pressure (mmHg)	129 ± 13	127 ± 14	126 ± 12.0
Diastolic blood pressure (mmHg)	81 ± 11	83 ± 10	81 ± 10
AST (IU/l)	26 ± 6.5	30 ± 11.9	32 ± 13.2
ALT (IU/l)	29 ± 12.1	38 ± 24.0	38 ± 24.7
LDH (IU/l)	315 ± 62.8	326 ± 56.0	344 ± 75.0
γ-GTP (IU/l)	82 ± 54.5	80 ± 66.5	85 ± 54.4
ALP (IU/l)	195 ± 52.3	203 ± 50.8	218 ± 53.9‡
Total protein (g/l)	72 ± 3.8	72 ± 3.7	72 ± 3.9
Creatinine (µmol/l)	84 ± 12.4	84 ± 13.3	86 ± 10.6
Total cholesterol (mmol/l)	5.5 ± 0.80	5.6 ± 0.95	5.3 ± 0.95
Triglycerides (mmol/l)	1.9 ± 1.12	1.9 ± 1.05	1.6 ± 0.90
HDL-C (mmol/l)	1.5 ± 0.32	1.4 ± 0.41	1.4 ± 0.22
LDL-C (mmol/l)	3.3 ± 0.61	3.3 ± 0.77	3.2 ± 0.84
Uric acid (mg/l)	61.7 ± 13.4	60.0 ± 11.3	59.0 ± 11.2
S-Amylase (IU/l)	88.5 ± 20.4	87.9 ± 20.6	101.3 ± 35.9
ESR (mm/h)	5.9 ± 5.8	6.8 ± 7.5	8.5 ± 7.8*‡
WBC (/µl)	6481 ± 1846	6222 ± 1873	6096 ± 1476
RBC (×10^4^/µl)	490.6 ± 35.7	486.8 ± 35.0	479.8 ± 40.7
Haemoglobin (g/dl)	15.4 ± 1.0	15.5 ± 0.9	15.3 ± 1.2
Haematocrit (%)	48.3 ± 2.7	48.6 ± 2.6	47.6 ± 3.7
FPG (mmol/l)	5.5 ± 0.5	5.7 ± 0.6	6.1 ± 0.6†§
1 h-glucose (mmol/l)	10.6 ± 2.2	11.1 ± 2.0	11.3 ± 2.1
2 h-glucose (mmol/l)	8.4 ± 0.8	8.7 ± 0.8	8.8 ± 1.3
Urine protein (+/−)	2/37	2/51	2/34
Fatty liver (+/−)	10/29	14/39	14/21
Night duty (+/−)	4/30	4/42	8/26
Blue-collar worker (+/−)	28/11*	48/5	27/9*
Administrative position (+/−)	0/39	0/53	1/35
Business bachelor (+/−)	3/36	7/46	10/26‡
Stress in daily life (+/−)	4/35	8/45	6/30
Satisfaction with lifestyle	5.13 ± 3.11	5.44 ± 2.38	6.49 ± 3.78
Egogram (CP > NP)	11/28	7/46	8/28
Egogram (AC > FC)	12/27	13/40	9/27
Fatigue (grade 1–4)	0.69 ± 1.03	1.00 ± 1.22	1.00 ± 1.22
Alcohol drinking (+/−)	36/3	41/12	33/3
Current smoking (+/−)	12/27	25/28	17/19

* *P* < 0.05;

† *P* < 0.01 (‘Persistent’ vs. ‘Recovered’ or ‘DM’);

‡ *P* < 0.05;

§ *P* < 0.01 (‘Recovered’ vs. ‘DM’).

AC, adapted child; ALP, alkaline phosphatase; ALT, alanine aminotransferase; AST, aspartate transaminase; CP, critical parent; DM, diabetes mellitus; ESR, erythrocyte sedimentation rate; FC, free child; FPG, fasting plasma glucose; γ-GTP, gamma-glutamyl transpeptidase; HDL-C, high-density lipoprotein cholesterol; IFG, impaired fasting glucose; IGT, impaired glucose tolerance; LDH, lactic dehydrogenase; LDL-C, low-density lipoprotein cholesterol; NP, nurturing parent; RBC, red blood cell; WBC, white blood cell. [‘Recovered’, participants with recovery of NGT from IFG and/or IGT at baseline during the observation period; ‘Persistent’, participants with persistent IFG and/or IGT; ‘DM’, participants with progression to DM from IFG and/or IGT.]

Next, multivariate analyses were performed to comprehensively determine independent risk factors for progression to DM from IFG and/or IGT, as well as factors associated with the recovery of NGT from IFG and/or IGT, by adjusting for age, BMI, systolic blood pressure, ALT, LDH, γ-GTP, ALP, TP, Cr, TG, HDL-C, LDL-C, UA, S-amylase, ESR, WBC, Hb, FPG, urinary protein, night duty, blue-collar job, administrative position, business bachelor, stress in daily life, satisfaction with lifestyle, Egogram, fatigue, alcohol drinking and smoking status. As shown in Table 2, night duty (*P* < 0.01), higher FPG within the range 6.1–6.9 mmol/l (*P* < 0.05), stress in daily life (*P* < 0.05) and having an administrative position (*P* < 0.05) were significant independent risk factors for the development of DM from IFG and/or IGT. Being a business bachelor did not reach statistical significance. In contrast, lower FPG, within the range 6.1–6.9 mmol/l (*P* < 0.05), having a white-collar job (*P* < 0.05), being a non-smoker (*P* < 0.05) and having lower serum ALT levels (*P* < 0.05) were significant independent factors for the recovery of NGT from IFG and/or IGT (Table 3). Lower systolic blood pressure did not reach significance.

**Table 3:** Multiple regression analysis of beneficial factors for the recovery of NGT from IFG and/or IGT

	*P*	HR	95% CI
FPG	0.017*	0.94	0.894–0.989
Blue-collar worker	0.033*	0.34	0.127–0.917
Smoker	0.040*	0.31	0.098–0.948
ALT	0.042*	0.97	0.947–0.999
Systolic blood pressure	0.085	0.86	0.724–1.021

* *P* < 0.05.

Model: adjusted for age, body mass index (BMI), systolic blood pressure, alanine aminotransferase (ALT), lactic dehydrogenase (LDH), gamma-glutamyl transpeptidase (γ-GTP), alkaline phosphatase (ALP), total protein, creatinine, triglyceride, high-density lipoprotein cholesterol (HDL-C), low-density lipoprotein cholesterol (LDL-C), uric acid, S-amylase, erythrocyte sedimentation rate (ESR), white blood cell (WBC), haemoglobin, FPG, urinary protein, night duty, blue-collar job, administrative position, business bachelor, stress in daily life, satisfaction with lifestyle, Egogram (CP > NP), Egogram (AC > FC), fatigue, alcohol drinking and current smoking.

AC, adapted child; CI, confidence interval; CP, critical parent; FC, free child; FPG, fasting plasma glucose; HR, hazard ratio; IFG, impaired fasting glucose; IGT, impaired glucose tolerance; NP, nurturing parent.

**Table 2:** Multiple regression analysis of risk factors for the progression to Type 2 diabetes from IFG and/or IGT

	*P*	HR	95% CI
Night duty	0.002*	5.48	1.82–16.49
FPG	0.031*	1.05	1.01–1.10
Stress in daily life	0.037*	3.81	1.09–13.35
Administrative position	0.045*	12.70	1.06–150.83
Business bachelor	0.064	2.68	0.95–7.57

* *P* < 0.05.

Model: adjusted for age, body mass index (BMI), systolic blood pressure, alanine aminotransferase (ALT), lactic dehydrogenase (LDH), gamma-glutamyl transpeptidase (γ-GTP), alkaline phosphatase (ALP), total protein, creatinine, triglyceride, high-density lipoprotein cholesterol (HDL-C), low-density lipoprotein cholesterol (LDL-C), uric acid, S-amylase, erythrocyte sedimentation rate (ESR), white blood cell (WBC), haemoglobin, FPG, urinary protein, night duty, blue-collar job, administrative position, business bachelor, stress in daily life, satisfaction with lifestyle, Egogram (CP > NP), Egogram (AC > FC), fatigue, alcohol drinking and current smoking.

AC, adapted child; CI, confidence interval; CP, critical parent; FC, free child; FPG, fasting plasma glucose; HR, hazard ratio; IFG, impaired fasting glucose; IGT, impaired glucose tolerance; NP, nurturing parent.

## Discussion

To comprehensively determine factors affecting the progression of Type 2 diabetes and the recovery of NGT from IFG and/or IGT, we conducted a study in which we prospectively investigated Japanese workers with IFG and/or IGT, who are at high risk of developing diabetes [14,15]. We found that, in addition to FPG levels, social and psychological factors such as status at work (night duty), stress in daily life and social position (administrative position) are also independent risk factors for the progression to DM from IFG and/or IGT, while being a white-collar worker and a non-smoker are factors associated with the recovery of NGT.

In our study, the rate of development of overt diabetes from IFG and/or IGT was equal to or relatively low (28.1%) and the rate (30.5%) of reversal of NGT was relatively high, compared with other reports [14–17]. There are several possible reasons for these differences: (i) participants were educated about dietary and exercise therapy once or twice a year by dieticians and medical doctors; (ii) participants were non-obese (mean BMI less than 25.0 kg/m^2^); and (iii) the DM group gained only a small amount of weight (less than 1 kg over 3.2 years), whereas the recovered group lost a small amount of weight, approximately 0.1 kg (data not shown). These factors might reduce the development of diabetes.

Furthermore, known risk factors such as obesity and elevated liver enzyme levels [1–5] were not found to be risk factors for the development of diabetes from IFG and/or IGT in our study, probably because the majority of participants analysed were not obese and they were already at the highest risk; i.e. they had IFG and/or IGT. Among participants with IFG and/or IGT at baseline, those with higher FPG levels (6.1–6.9 mmol/l) might have greater impairment of insulin secretion than those with lower FPG levels, which were associated with the recovery of NGT from IFG and/or IGT.

The social and psychological factors, such as night duty, stress in daily life and administrative position, resulted in the progression from IFG and/or IGT to overt diabetes, possibly as a result of increasing insulin resistance. In our study, night duty was the highest risk factor for progression to diabetes. Subjects who are on night duty experience sleep disorders, which are known to affect the sympathetic nervous system and to be associated with impaired glucose tolerance [24]. In addition, short sleep duration is a risk factor for developing diabetes, independent of confounding factors [25]. Furthermore, stress in daily life and status at work (administrative position) were risk factors for the development of diabetes. Persons in administrative positions often experience both physical and mental stress [26]. In our case, because they were middle managers, they may have experienced strong job strain and stress as a result of high job demands combined with low job decision latitude and effort–reward imbalance, factors which are associated with Type 2 diabetes [7–11] and cardiovascular diseases [27–29].

It has been proposed that stress activates the hypothalamo–pituitary–adrenal axis and the central sympathetic system and leads to the development of endocrine perturbation and obesity, which increases insulin resistance, causing Type 2 diabetes [30,31]. In addition, increased levels of stress hormones such as catecholamines and glucocorticoids may impair insulin secretion [32]. Furthermore, stress may induce pro-inflammatory cytokines [33] and DNA damage [34], factors which are related to Type 2 diabetes.

Interestingly, approximately one-third (30.5%) of participants with IFG and/or IGT at baseline returned to NGT. A multivariate analysis indicated that baseline factors related to improvement of glucose intolerance also included social factors (white-collar worker) and lifestyle (non-smoking), as well as lower levels of FPG (6.1–6.9 mmol/ll) and serum ALT levels. Smoking is a risk factor for IGT and Type 2 diabetes [35,36], while serum ALT level is a risk factor for Type 2 diabetes [3,4]. Therefore, non-smoking and low serum ALT levels may be associated with a return to NGT from IFG and/or IGT.

Although this is one of the only studies to prospectively examine relationships between psychosocial factors and risk of diabetes, further study is necessary. One limitation of this study is that many variables were examined with only a small sample size.

In summary, the results of the present study indicate that, in addition to glucose levels, social and psychological factors also affect progression to Type 2 diabetes or recovery of NGT from IFG and/or IGT in Japanese workers and social and psychological interventions may need to be considered to prevent the development of Type 2 diabetes in those at high risk.

## Competing interests

Nothing to declare.

## Abbreviations

ALP: alkaline phosphatase
ALT: alanine aminotransferase
BMI: body mass index
Cr: creatinine
DM: diabetes mellitus
ESR: erythrocyte sedimentation rate
FPG: fasting plasma glucose
γ-GTP: gamma-glutamyl transpeptidase
Hb: haemoglobin
HDL-C: high-density lipoprotein cholesterol
IFG: impaired fasting glucose
IGT: impaired glucose tolerance
LDH: lactic dehydrogenase
LDL-C: low-density lipoprotein cholesterol
NGT: normal glucose tolerance
OGTT: oral glucose tolerance test
TG: triglyceride
TP: total protein
UA: uric acid
WBC: white blood cell
